# AI as a peer reviewer: a blinded comparative study of LLM-generated and human reviews in a cardiology journal

**DOI:** 10.1093/ehjimp/qyag097

**Published:** 2026-05-27

**Authors:** Edoardo Zancanaro, Andreas Giannopoulos, Alessia Gimelli, Karl-Patrik Kresoja

**Affiliations:** Brigham and Women's Hospital, Harvard Medical School, 45 st francis st., Boston, MA 02115, USA; Department of Nuclear Medicine, Cardiac Imaging, University Hospital Zurich, Zürich, Switzerland; Imaging Department, Fondazione CNR-Regione Toscana ‘Gabriele Monasterio’, Pisa, Italy; Department of Cardiology, Cardiology I, University Medical Center Mainz, Langenbeckstraße 1, 55131 Mainz, Germania

**Keywords:** artificial intelligence, large language models, peer review, cardiology, editorial decision

## Abstract

**Aims:**

Peer review is a cornerstone of scientific quality control, yet it is increasingly burdened by growing manuscript volumes and reviewer fatigue. Large language models (LLMs) have emerged as potential tools to support scientific review, but it remains unclear whether AI-generated reviews are equivalent to human reviews on the endpoint that ultimately matters, agreement with the final editorial decision.

**Methods and results:**

We retrieved 40 manuscripts previously submitted to a cardiology journal (20 ultimately accepted, 20 *de novo* rejected) along with all available historical human peer reviews (*n* = 77). For each manuscript, we generated a corresponding peer review using LLM in deep research mode (*n* = 41). All 118 reviews were reformatted into a single anonymous template by two unblinded investigators and scored independently by two blinded editors across seven domains (digestion, focus, balance, suggestions, precision, politeness, and conclusiveness; 0–2 scale). The primary endpoint was concordance between each reviewer recommendation (in favour of vs. against publication) and the final editorial decision. Secondary endpoints were domain-specific quality scores and AI–human inter-rater agreement (Cohen’s κ). Concordance with the final editorial decision was 67.5% for AI-generated reviews (27/40) and 71.9% for the human consensus (23/32 evaluable; *P* = 0.74). Stratified by editorial outcome, AI correctly recommended publication in 75% of accepted manuscripts and rejection in 60% of rejected manuscripts; the corresponding figures for the human consensus were 88% and 56%. AI-generated reviews scored significantly higher than human reviews in five of seven quality domains (focus, balance, suggestions, precision, and conclusiveness; all *P* < 0.05), with a higher total sum score (13.2 ± 0.9 vs. 11.4 ± 2.0; *P* < 0.001). AI–human inter-rater agreement was substantial (κ = 0.73), exceeding human–human agreement on the same articles (κ = 0.54). AI reviews were generated in 2–6 min vs. a median 17-day turnaround for human reviews.

**Conclusion:**

LLM-generated peer reviews are non-inferior to human reviews in terms of agreement with the final editorial decision, while showing higher internal consistency, comparable quality on structured domains, and substantially shorter turnaround. These findings support the integration of AI as a complementary tool in editorial workflows, rather than as a replacement for human peer review.

## Central Illustration

**Central Illustration:**
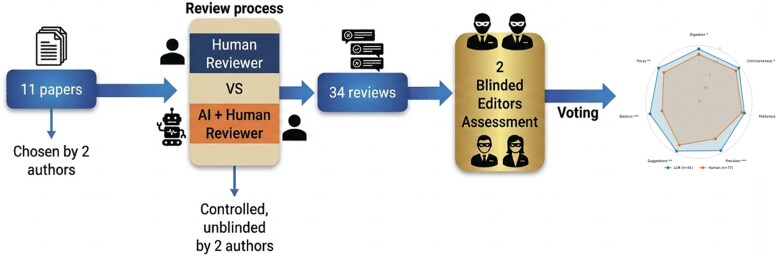
Schematic of the study workflow.

## Introduction

Peer review represents the gold standard for evaluating scientific manuscripts prior to publication, yet the system is under considerable strain. The exponential growth of scientific output has outpaced the availability of qualified reviewers, resulting in longer turnaround times, reviewer fatigue, and concerns about the consistency and quality of reviews.^1,2^ In cardiology, where methodological rigour and clinical relevance are paramount, high-quality and timely peer review is particularly critical.^3^

The advent of large language models (LLMs), exemplified by OpenAI'’s ChatGPT, has opened new possibilities for automating or augmenting complex cognitive tasks in medicine and research.^4,5^ Several reports have demonstrated that LLMs can generate coherent, detailed feedback on scientific manuscripts,^6,7^ and recent comparative studies in surgery^8^ and educational research^9^ have suggested broad parity in review quality between AI and human reviewers. However, prior comparisons have largely focused on the quality of the review as a written product, while the metric that ultimately matters in editorial practice, whether the reviewer’s conclusion is in line with the final decision on the fate of the manuscript, has rarely been examined.

We therefore performed a blinded, head-to-head comparison between LLM-generated reviews and historical human peer reviews of manuscripts submitted to a cardiology journal, using both a structured multi-domain quality framework and, as the primary endpoint, concordance with the final editorial decision.

## Methods

### Study design and manuscript selection

We retrieved 40 consecutive manuscripts previously submitted to a cardiology journal (EHJ-IMP), selected to provide a balanced design with 20 articles ultimately accepted (after revision) and 20 articles *de novo* rejected, based on the final editorial verdict. No manuscript was excluded based on perceived review quality to preserve external validity. For these 40 manuscripts, 77 historical human peer reviews from the first evaluation round were available; reviews from revision rounds were not used.

### LLM review generation

LLM system (Claude, ChatGPT, and Gemini) in deep research mode, accessed through the standard web interface, was used to generate the AI reviews, after extensive training and mode used.^10^ Each manuscript was uploaded in full to the model with a single standardized prompt instructing it to perform a thorough peer review covering methodology, data integrity, clinical relevance, and manuscript quality, following the standards of a high-impact cardiology journal. The prompt was identical across all sessions. One AI review was generated per manuscript (*n* = 40); for one randomly selected manuscript, a second independent AI review was generated 1 week later as a pilot test of within-LLM reproducibility (total AI reviews *n* = 41). Generation time per review ranged from 2 to 6 min.

### Reformatting and blinding

Two investigators, fully aware of the origin of each review, manually reformatted all 118 reviews into a single homogeneous template. The reformatting included removal of bullet-point structures, normalization of section headings, breakage of overly systematic enumerations, and rephrasing of stylistic markers commonly associated with LLM output (e.g. ‘Strengths/Weaknesses’ headers, hierarchical numbered lists, formulaic openings). The aim was to neutralize, as far as possible without altering the content, stylistic cues that might have allowed the editors to recognize the origin of the review. The reformatted reviews were then distributed to two independent, blinded evaluators, experienced editors, with no knowledge of each review’s origin.

### Quality assessment framework

Each review was scored on a three-point scale (0 = insufficient, 1 = partial, 2 = sufficient) across seven pre-specified quality domains: (i) digestion (overall comprehension of the manuscript), (ii) focus (relevance of comments to the manuscript’s core content), (iii) balance (fairness in addressing strengths and weaknesses), (iv) suggestions for improvement (actionability of the proposed revisions), (v) precision (specificity and accuracy of critique), (vi) politeness (professional and respectful tone), and (vii) conclusiveness (clarity of the overall editorial recommendation). A total sum score (range 0–14) was also calculated. We deliberately retained a coarse scale, given the intrinsically categorical nature of the domains (insufficient/partial/sufficient) and the limited number of evaluators, but acknowledge in the Discussion the trade-off involved (see Limitations).

### Concordance with editorial decision (primary endpoint)

Each reviewer’s recommendation (accept, minor revision, major revision, *de novo*, reject) was dichotomized into ‘in favour of publication’ (any non-reject recommendation) vs. ‘against publication’ (reject). For each review, we computed the agreement between this binary recommendation and the final editorial decision (accept/reject). At the article level, we additionally computed the human consensus by majority vote across the human reviewers of each article (articles with a tied human vote were excluded from the article-level analysis but retained for review-level analyses).

### Statistical analysis

Continuous variables are reported as mean ± standard deviation. Domain scores were compared between AI and human reviews using the Mann–Whitney U test, given the ordinal nature of the data. Categorical variables (concordance with editorial decision, reviewer recommendations) were compared using Fisher’s exact test or the χ^2^ test as appropriate. Inter-rater agreement was quantified using Cohen’s κ. A two-sided *P*-value <0.05 was considered statistically significant. Analyses were performed using SPSS v.28 (IBM, Armonk, NY, USA) and Python 3.11 (SciPy 1.11).

## Results

### Dataset

A total of 118 reviews of 40 manuscripts were evaluated: 41 generated by an LLM and 77 written by human reviewers. The number of human reviewers per manuscript ranged from 1 to 3 (median 2). The distribution of recommendations is summarized in *Table 1*: AI reviews uniformly fell into either major revision (24/41, 59%) or reject (17/41, 41%); human reviews were more heterogeneously distributed across reject (28/77, 36%), major revision (38/77, 49%), minor revision (8/77, 10%), *de novo* (2/77, 3%), and accept (1/77, 1%).

**Table 1:** Quality scores of LLM-generated vs. human peer reviews across seven domains

Domain	LLM (*n* = 41)	Human (*n* = 77)	*P*-value
Digestion	1.88 ± 0.33	1.69 ± 0.49	0.031*
Focus	1.93 ± 0.26	1.68 ± 0.47	0.002*
Balance	1.88 ± 0.33	1.42 ± 0.52	<0.001*
Suggestions	1.93 ± 0.26	1.69 ± 0.52	0.007*
Precision	1.90 ± 0.30	1.44 ± 0.50	<0.001*
Politeness	1.76 ± 0.43	1.66 ± 0.48	0.30
Conclusiveness	1.93 ± 0.26	1.78 ± 0.42	0.043*
**Total sum score**	**13.20** ± **0.90**	**11.35** ± **2.03**	**<0**.001*

Data are mean ± SD.

LLM = large language model.

^*^ *P* < 0.05 (Mann–Whitney U test).

### Concordance with the editorial decision

**Figure 1:**
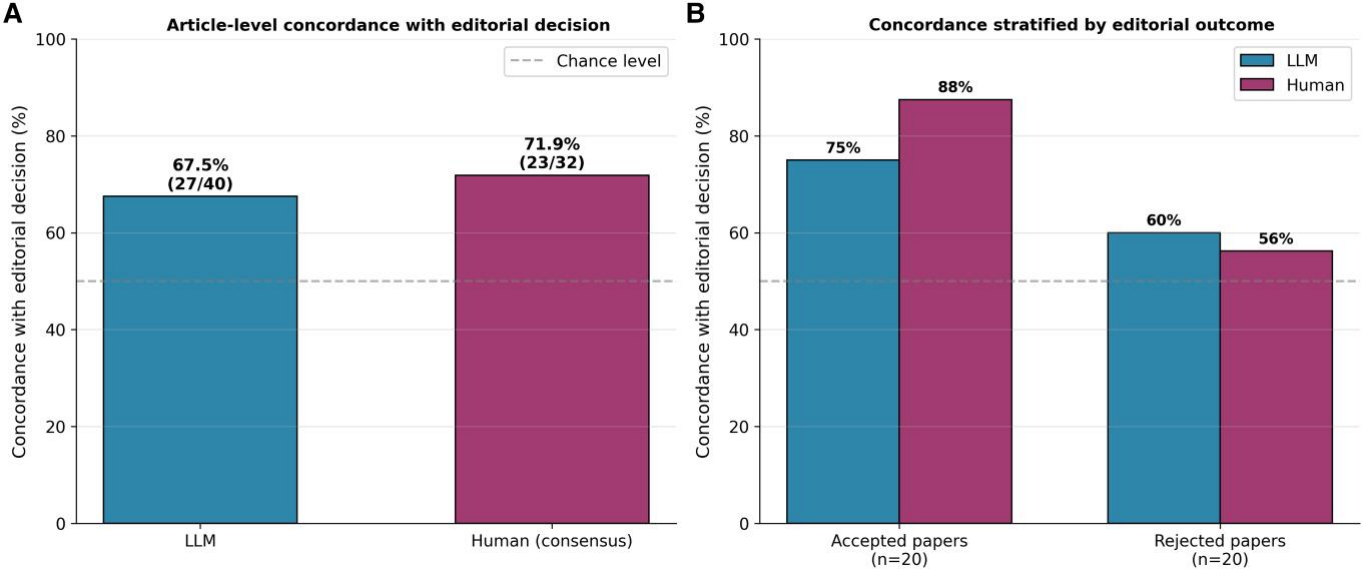
Concordance with the final editorial decision. (*A*) Article-level concordance for AI-generated reviews and the human consensus (majority vote across human reviewers; ties excluded). (*B*) Concordance stratified by editorial outcome (accepted vs. rejected manuscripts).

**Figure 2:**
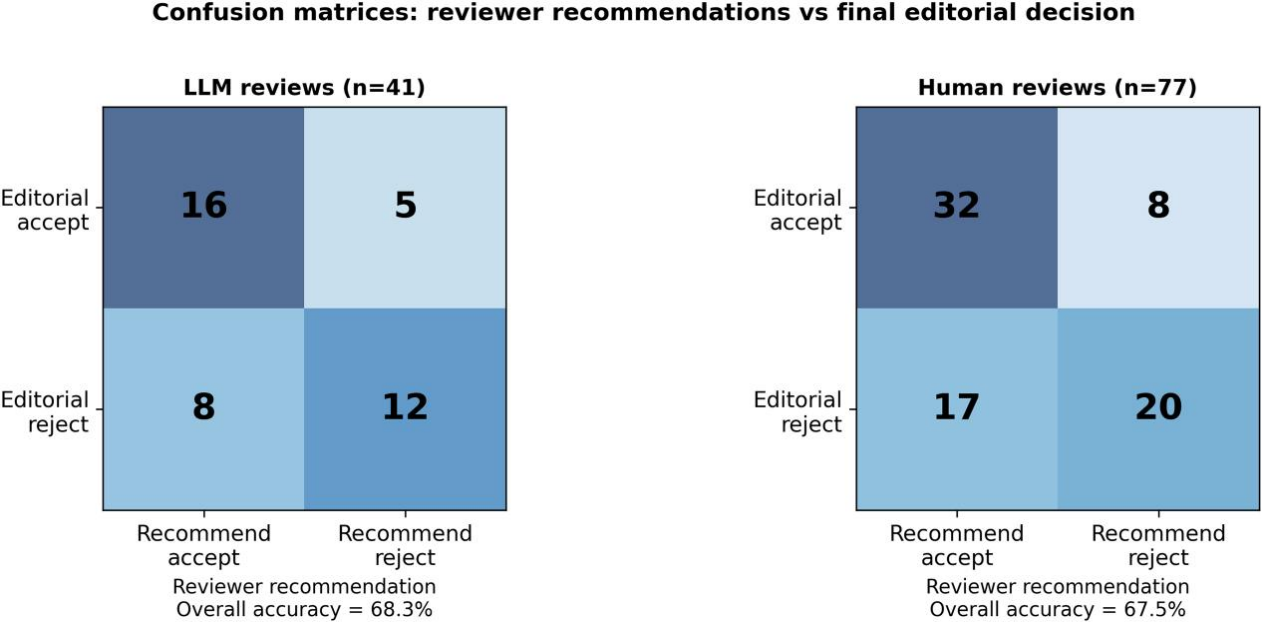
Confusion matrices comparing reviewer recommendations (in favour vs. against publication) with the final editorial decision, separately for AI-generated reviews and for human reviews.

At the review level, AI-generated reviews matched the final editorial decision in 28/41 cases (68.3%) and human reviews in 52/77 cases (67.5%; *P* = 1.00). At the article level, AI matched in 27/40 (67.5%); the human consensus matched in 23/32 evaluable articles (71.9%; *P* = 0.74) (*Figure 1A*). Stratified by editorial outcome (*Figure 1B*), AI correctly recommended publication in 15/20 accepted articles (75%) and rejection in 12/20 rejected articles (60%); the corresponding figures for the human consensus were 14/16 (88%) and 9/16 (56%). Both groups thus showed a similar pattern, with greater accuracy on accepted than on rejected manuscripts, and overall comparable performance. Confusion matrices for AI and human reviews are provided in *Figure 2*, *Table 2*.

**Table 2:** Concordance between reviewer recommendations and the final editorial decision

Metric	LLM	Human	*P*-value
Review-level concordance	28/41 (68.3%)	52/77 (67.5%)	1.00
Article-level concordance	27/40 (67.5%)	23/32 (71.9%)	0.74
Concordance, accepted papers	15/20 (75%)	14/16 (88%)	0.42
Concordance, rejected papers	12/20 (60%)	9/16 (56%)	1.00
AI–Human Cohen’s κ	0.73 (substantial)	—	—
Human–Human Cohen’s κ	—	0.54 (moderate)	—
Median time per review	2–6 min	17 days	<0.001^a^

Reviewer recommendations were dichotomized as ‘in favour of publication’ (accept, minor revision, major revision, *de novo*) vs. ‘against publication’ (reject). Article-level human consensus by majority vote; ties excluded.

LLM = large language model.

^a^Wall-clock turnaround.

Bottom line, all the articles analysed (accepted and rejected *de novo*) have been published.

### Quality domain scores

**Figure 3:**
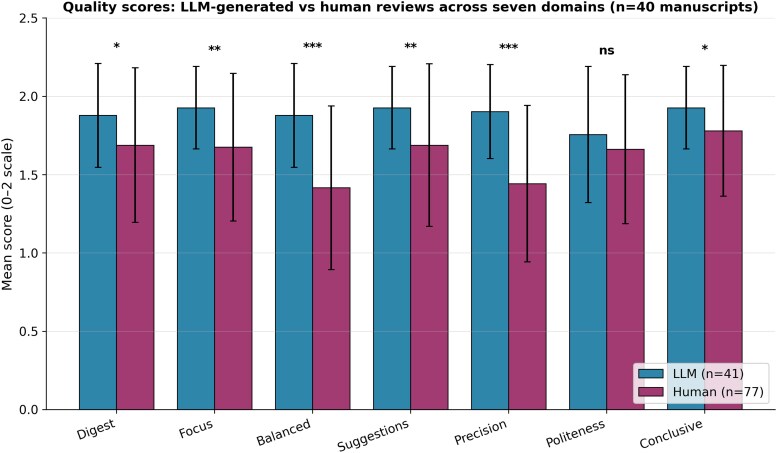
Quality scores of LLM-generated vs. human peer reviews across the seven structured domains. Bars show mean ± SD; *P < 0.05, **P < 0.01, ***P < 0.001 (Mann–Whitney U test); ns = not significant.

**Figure 4:**
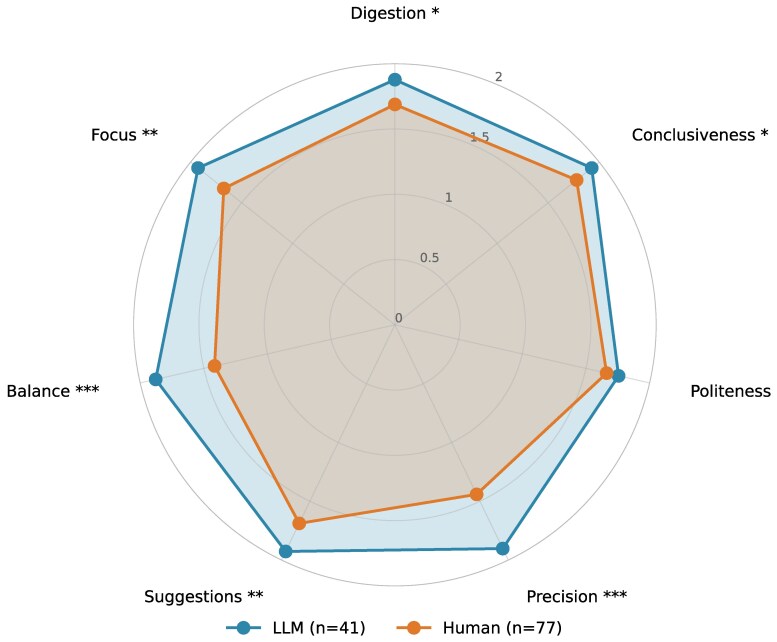
Radar plot on quality scores of LLM-generated vs. human peer reviews across the seven structured domains.

AI-generated reviews scored higher than human reviews in five of seven domains: focus (1.93 ± 0.26 vs. 1.68 ± 0.47; *P* = 0.002), balance (1.88 ± 0.33 vs. 1.42 ± 0.52; *P* < 0.001), suggestions (1.93 ± 0.26 vs. 1.69 ± 0.52; *P* = 0.007), precision (1.90 ± 0.30 vs. 1.44 ± 0.50; *P* < 0.001), and conclusiveness (1.93 ± 0.26 vs. 1.78 ± 0.42; *P* = 0.043). Differences in digestion (1.88 ± 0.33 vs. 1.69 ± 0.49; *P* = 0.031) were modest, while politeness was indistinguishable (1.76 ± 0.43 vs. 1.66 ± 0.48; *P* = 0.30). The total sum score was higher for AI reviews (13.20 ± 0.90 vs. 11.35 ± 2.03; *P* < 0.001) (*Figures 3* and *4*, *Table 1*). The pattern was consistent across both accepted and rejected manuscripts (see Supplementary data online, *Table S1 and S2*).

### Inter-rater agreement

AI–human inter-rater agreement on the binary recommendation was substantial (κ = 0.73; 87.2% raw agreement across 78 paired comparisons). Human–human agreement on the same articles was lower (κ = 0.54; 80.0% raw agreement across 40 paired comparisons), suggesting that AI reviews are not only as accurate as human reviews on the editorial endpoint but also more internally consistent (see Supplementary data online, *Figure S1*).

### Time to review

Wall-clock turnaround time for human reviews, retrieved from the editorial system, ranged from 6 to 41 days (median 17). AI reviews were generated in 2–6 min per manuscript. While wall-clock time is not equivalent to active reviewing time, this difference of approximately three orders of magnitude is operationally relevant for editorial workflow planning.

## Discussion

In a balanced cohort of 40 manuscripts (20 accepted, 20 *de novo* rejected) and 118 peer reviews from a cardiology journal, AI-generated reviews matched the final editorial decision at a rate statistically indistinguishable from that of human reviews (67.5% vs. 71.9%), while showing higher quality scores on five of seven structured domains, substantially higher inter-rater agreement, and orders-of-magnitude shorter turnaround. We interpret these findings as evidence of non-inferiority of AI peer review on the metric that ultimately matters most for editors, agreement with the final decision, rather than as evidence that AI ‘outperforms’ human reviewers.

Our findings extend prior work on AI-assisted peer review in surgery^8^ and education^9^ by anchoring the comparison on a clinically meaningful outcome. The observation that both AI and human reviewers were more accurate on accepted than on rejected manuscripts is intuitive and reflects a well-known reviewer leniency bias: it is cognitively easier to identify the strengths of a sound paper than to confidently recommend rejection of a flawed one. Importantly, this bias was present in both groups, suggesting it is not specific to AI.

The substantially higher AI–human inter-rater agreement (κ = 0.73) compared with human–human agreement (κ = 0.54) deserves comment. It implies that the ‘noise’ between reviewers, long recognized as a weakness of peer review,^1^ is reduced, not amplified, when one of the two reviewers is an LLM. This is consistent with our domain-level observations: AI reviews show very low variance on balance, suggestions, and conclusiveness, qualities that are difficult to maintain consistently for human reviewers facing fatigue and time pressure.^2^

Several limitations should be acknowledged. First, although we devoted considerable effort to neutralize stylistic cues through manual reformatting, we cannot fully exclude that residual features still allowed the blinded editors to recognize AI-origin reviews. The likely direction of any such ‘novelty bias’ would be in favour of the new product (AI), and our key concordance endpoint, which is independent of editor perception, is robust to this bias. Second, we used a coarse three-point scale to score quality domains; a finer scale might yield different results, although the central concordance analysis is scale independent. Third, only two evaluators were used, and inter-rater variability between them was not formally assessed; this is a deliberate trade-off favouring evaluator expertise over breadth and is mitigated by the larger article and review counts. Fourth, the LLM used represents a snapshot in time; both quality and detectability are likely to evolve rapidly. Fifth, the dataset comes from a single journal, and external validity to other fields and to other LLMs remains to be tested.^11,12^

We deliberately did not select only ‘high-quality’ historical human reviews. A restriction of that kind would have introduced a strong selection bias in favour of human reviewers and would have undermined the external validity of any concordance analysis: editors do not, in everyday practice, receive only best-in-class reviews. By keeping the human reviews unfiltered, we benchmarked AI against the operationally relevant comparator.

Our results have practical implications for editorial workflow. Even setting aside the question of whether AI reviews should replace human ones, which we do not advocate, the combination of high concordance with editorial decisions, high consistency, and short turnaround makes AI-assisted review a plausible candidate for triage, second-opinion, and quality-floor functions. A polite and balanced human review that ultimately recommends acceptance of a flawed manuscript, as the reviewer of an earlier version of this work rightly noted, has not done a valuable job; in our data, AI reviews are no more prone to this failure mode than the average human review, and arguably less so on rejected papers.

## Next step

The purpose of this small study was to create a proof of concept to understand the feasibility. The next step will be a larger scale of papers and more heterogeneous reviewers possible also in the context of a randomized design.

## Conclusions

LLM-generated peer reviews are non-inferior to human reviews in their agreement with the final editorial decision in cardiology manuscripts, while offering higher consistency, comparable quality on structured domains, and substantially shorter turnaround. These results support a complementary, rather than replacement, role for AI in editorial peer review and call for prospective, multi-journal studies and for clear editorial policies governing the use of AI in this setting.

## Supplementary Material

qyag097_Supplementary_Data

## Lead author biography

Edoardo Zancanaro, a cardiac surgery resident at San Raffaele Hospital in Milan, Italy, and graduate of the International Medical Program at Università Vita-Salute San Raffaele, joins the EACTS Council as Residents’ Representative. He has recently completed an advanced fellowship in Minimally Invasive Cardiac Surgery and Interventional Cardiology at the University Hospital Mainz, Germany, and he is now pursuing a fellowship in Minimally Invasive Cardiac Surgery and Artificial Intelligence Science at Brigham and Women’s Hospital and Harvard Medical School in Boston, USA, respectively. With a focus on valvular heart disease, minimally invasive/robotic cardiac surgery, and transcatheter valve intervention, Dr Zancanaro is part of a new generation of surgeons combining clinical excellence with technological innovation. Passionate about education, he also leads podcasts and broadcasts for EHJ and the ESC (IMPact podcast) and the new EACTS broadcast, helping share knowledge and inspire fellow trainees. As the Residents’ Representative on the EACTS Council, he is dedicated to championing the voices of residents, interns, and fellows, fostering collaboration, and ensuring the next generation has a strong platform within the Association, as well as preparing them for new frontiers like AI, MedTech, and more.
