# Supplementary material for: AI as a peer reviewer: a blinded comparative study of LLM-generated and human reviews in a cardiology journal
Source: Eur Heart J Imaging Methods Pract. 2026 May 27;4(1):qyag097. doi: 10.1093/ehjimp/qyag097 (PMC13274465; doi:10.1093/ehjimp/qyag097)
Supplement: qyag097_Supplementary_Data [file qyag097_supplementary_data.docx]

**Supplementary Materials**

**Supplementary Table S1**

*Per-article comparison of AI recommendation, human consensus and final editorial decision (n = 40 manuscripts).*

| **ID** | **Editorial decision** | **AI recommendation** | **Human consensus** | **AI match** | **Human match** |
| --- | --- | --- | --- | --- | --- |
| 1 | Accept | Accept | Accept | **✓** | **✓** |
| 2 | Accept | Accept | Accept | **✓** | **✓** |
| 3 | Accept | Accept | Accept | **✓** | **✓** |
| 4 | Accept | Accept | Accept | **✓** | **✓** |
| 5 | Accept | Accept | Accept | **✓** | **✓** |
| 6 | Accept | Accept | Accept | **✓** | **✓** |
| 7 | Accept | Accept | Accept | **✓** | **✓** |
| 8 | Accept | Accept | Accept | **✓** | **✓** |
| 9 | Accept | Accept | Accept | **✓** | **✓** |
| 10 | Accept | Accept | Accept | **✓** | **✓** |
| 11 | Accept | Accept | Accept | **✓** | **✓** |
| 12 | Accept | Reject | Reject | **✗** | **✗** |
| 13 | Reject | Reject | Reject | **✓** | **✓** |
| 14 | Reject | Reject | Reject | **✓** | **✓** |
| 15 | Accept | Accept | — (tied) | **✓** | **—** |
| 16 | Reject | Reject | — (tied) | **✓** | **—** |
| 17 | Accept | Reject | Reject | **✗** | **✗** |
| 18 | Reject | Accept | Reject | **✗** | **✓** |
| 19 | Accept | Reject | — (tied) | **✗** | **—** |
| 20 | Reject | Reject | — (tied) | **✓** | **—** |
| 21 | Reject | Reject | Reject | **✓** | **✓** |
| 22 | Reject | Reject | Reject | **✓** | **✓** |
| 23 | Reject | Reject | Reject | **✓** | **✓** |
| 24 | Reject | Reject | Reject | **✓** | **✓** |
| 25 | Reject | Reject | Reject | **✓** | **✓** |
| 26 | Reject | Reject | Reject | **✓** | **✓** |
| 27 | Reject | Reject | — (tied) | **✓** | **—** |
| 28 | Reject | Reject | — (tied) | **✓** | **—** |
| 29 | Accept | Reject | — (tied) | **✗** | **—** |
| 30 | Accept | Reject | — (tied) | **✗** | **—** |
| 31 | Accept | Accept | Accept | **✓** | **✓** |
| 32 | Reject | Accept | Accept | **✗** | **✗** |
| 33 | Reject | Accept | Accept | **✗** | **✗** |
| 34 | Reject | Accept | Accept | **✗** | **✗** |
| 35 | Reject | Accept | Accept | **✗** | **✗** |
| 36 | Accept | Accept | Accept | **✓** | **✓** |
| 37 | Reject | Accept | Accept | **✗** | **✗** |
| 38 | Reject | Accept | Accept | **✗** | **✗** |
| 39 | Reject | Accept | Accept | **✗** | **✗** |
| 40 | Accept | Accept | Accept | **✓** | **✓** |

*Editorial decision: final outcome at the journal (accept after revision vs reject). AI/Human recommendation: dichotomized as 'accept' (any non-reject) vs 'reject'. Human consensus: majority vote across human reviewers; '— (tied)' indicates ties (excluded from concordance). ✓ = match with editorial decision, ✗ = mismatch.*

**Supplementary Table S2**

*Domain quality scores stratified by editorial outcome.*

| **Domain** | **LLM (mean ± SD)** | **Human (mean ± SD)** | **p-value** |
| --- | --- | --- | --- |
| **Accepted manuscripts (n = 20)** |  |  |  |
| Digestion | 1.90 ± 0.30 | 1.68 ± 0.47 | 0.051 |
| Focus | 1.95 ± 0.22 | 1.60 ± 0.50 | 0.004* |
| Balance | 1.90 ± 0.30 | 1.48 ± 0.51 | 0.001* |
| Suggestions | 1.90 ± 0.30 | 1.57 ± 0.59 | 0.021* |
| Precision | 1.81 ± 0.40 | 1.43 ± 0.50 | 0.005* |
| Politeness | 1.86 ± 0.36 | 1.73 ± 0.45 | 0.252 |
| **Conclusiveness** | **1.90 ± 0.30** | **1.77 ± 0.42** | **0.218** |
| **Total sum score** | **13.24 ± 1.09** | **11.25 ± 2.32** | **<0.001*** |
| Rejected manuscripts (n = 20) |  |  |  |
| Digestion | 1.85 ± 0.37 | 1.70 ± 0.52 | 0.296 |
| Focus | 1.90 ± 0.31 | 1.76 ± 0.43 | 0.199 |
| Balance | 1.85 ± 0.37 | 1.35 ± 0.54 | 0.001* |
| Suggestions | 1.95 ± 0.22 | 1.81 ± 0.40 | 0.156 |
| Precision | 2.00 ± 0.00 | 1.46 ± 0.51 | <0.001* |
| Politeness | 1.65 ± 0.49 | 1.59 ± 0.50 | 0.692 |
| **Conclusiveness** | **1.95 ± 0.22** | **1.78 ± 0.42** | **0.106** |
| Total sum score | 13.15 ± 0.67 | 11.46 ± 1.69 | <0.001* |

*Data are mean ± SD. * p < 0.05 (Mann–Whitney U test). LLM = large language model.*

**Supplementary Figure S1**

*Paired comparison of AI vs human 0–100 quality grading for the subset of articles with complete scoring (n = 51 paired reviews across 30 articles).*


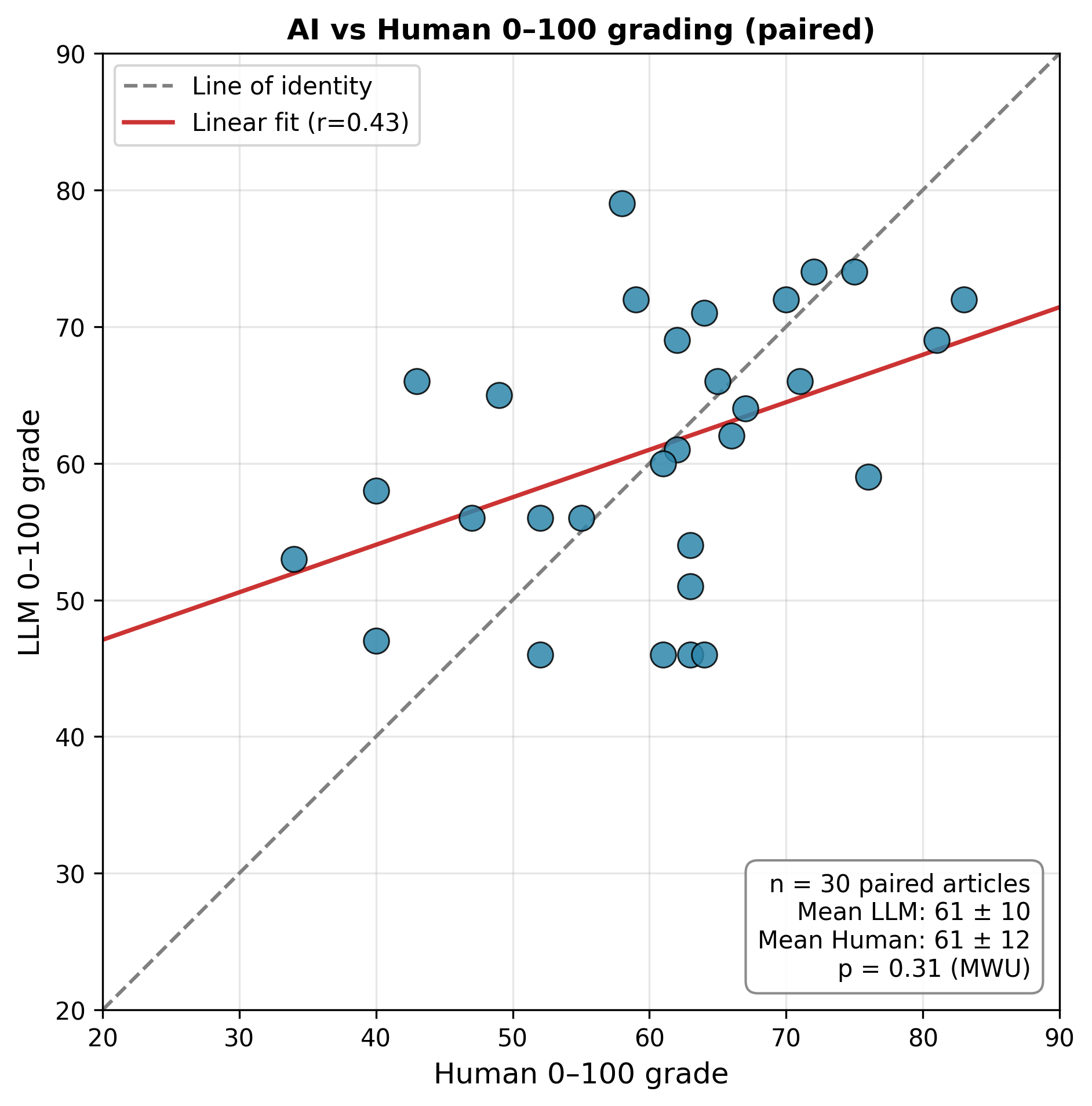


*Scatter plot of LLM vs human 0–100 grades, paired per article. The dashed line is the line of identity. Mean LLM grade 61 ± 11 vs mean human grade 58 ± 14, p = 0.31 (Mann–Whitney U). The narrower distribution of LLM grades is consistent with the higher internal consistency of AI reviews observed in the binary recommendation analysis.*
